# Beam flatness modulation for a flattening filter free photon beam utilizing a novel direct leaf trajectory optimization model

**DOI:** 10.1002/acm2.12837

**Published:** 2020-03-16

**Authors:** Nicholas J. Potter, Guanghua Yan, Hongcheng Liu, Haitham Alahmad, Darren L. Kahler, Chihray Liu, Jonathan G. Li, Bo Lu

**Affiliations:** ^1^ Department of Radiation Oncology College of Medicine University of Florida Gainesville FL USA; ^2^ Department of Industrial & Systems Engineering College of Engineering University of Florida Gainesville FL USA

**Keywords:** direct aperture, flattening filter free, IMRT, optimization, sliding window

## Abstract

Flattening filter free (FFF) linear accelerators produce a fluence distribution that is forward peaked. Various dosimetric benefits, such as increased dose rate, reduced leakage and out of field dose has led to the growth of FFF technology in the clinic. The literature has suggested the idea of vendors offering dedicated FFF units where the flattening filter (FF) is removed completely and manipulating the beam to deliver conventional flat radiotherapy treatments. This work aims to develop an effective way to deliver modulated flat beam treatments, rather than utilizing a physical FF. This novel optimization model is an extension of the direct leaf trajectory optimization (DLTO) previously developed for volumetric modulated radiation therapy (VMAT) and is capable of accounting for all machine and multileaf collimator (MLC) dynamic delivery constraints, using a combination of linear constraints and a convex objective function. Furthermore, the tongue and groove (T&G) effect was also incorporated directly into our model without introducing nonlinearity to the constraints, nor nonconvexity to the objective function. The overall beam flatness, machine deliverability, and treatment time efficiency were assessed. Regular square fields, including field sizes of 10 × 10 cm^2^ to 40 × 40 cm^2^ were analyzed, as well as three clinical fields, and three arbitrary contours with "concave" features. Quantitative flatness was measured for all modulated FFF fields, and the results were comparable or better than their open FF counterparts, with the majority having a quantitative flatness of less than 3.0%. The modulated FFF beams, due to the included efficiency constraint, were able to achieve acceptable delivery time compared to their open FF counterpart. The results indicated that the dose uniformity and flatness for the modulated FFF beams optimized with the DLTO model can successfully match the uniformity and flatness of their conventional FF counterparts, and may even provide further benefit by taking advantage of the unique FFF beam characteristics.

## 1 INTRODUCTION

The bremsstrahlung distribution of megavoltage x rays produced by the target in a medical linear accelerator (Linac) is a strongly forward peaked intensity distribution. The variation in both energy and intensity across the beamline is compensated for by introducing a flattening filter (FF) in the beamline. The physical filter is developed to produce a nominally flat beam at a designated depth below the patient surface to accommodate the uniform dose requirement of conventional treatments. However, the FF design itself has some major drawbacks for dosimetry. Studies have shown that the FF contributes to the majority of treatment head scatter, which results in an increase in patient skin dose.^1^, ^2^ It is also energy‐dependent and machine‐type‐dependent, which is not ideal for machine design, dosimetry, and treatment planning modeling.^1^, ^2^ More importantly, introducing the FF into the beam path significantly decreases the original dose rate due to the attenuating effect, and subsequently increases the beam delivery time. In the 1990s, several groups studied flattening filter free (FFF) high‐energy photon beams. The main focus for using FFF beams at that time was on boosting the dose rate for radiosurgery since dose uniformity is not a concern for radiosurgery treatment.^3^ In addition to the dose rate increase, it was concluded that FFF beams offer some dosimetric advantages compared to FF beams, such as lower out‐of‐field dose, lower head scatter magnitude, and less spectrum variation for various field sizes.^4^, ^5^, ^6^, ^7^, ^8^, ^9^, ^10^ Those features can help simplify the beam model and ultimately improve dose calculation accuracy.^11^, ^12^

In recent years, the focus of FFF studies and application has shifted to intensity modulated radiotherapy (IMRT) treatment techniques, as intensity modulation (IM) techniques inherently do not require a flat beam profile for beam modulation, and the dosimetric advantages of the FFF beam can be utilized in IMRT planning.^11^, ^13^ All contemporary linear accelerators now offer the options for beam delivery using both FF and FFF modes. This allows the user to choose either mode to tailor to various treatment techniques. For example, users can choose FF beams to deliver 2D/3D plans for conventional treatment and choose FFF beams for arc‐therapy and/or IMRT delivery. However, the preservation of both modes not only complicates the gantry head design, it also increases the workload for machine maintenance as well as for initial commissioning and routine quality assurance (QA). Hypothetically, if all of the current treatment delivery techniques could be facilitated using FFF beams, it would be ideal to completely remove the FF from the gantry head, which would simplify both the machine design and the physics/engineering QA. Nevertheless, the realization is that the removal of the FF from the machine would also restrict the capability to deliver conventional treatments that require flat beam delivery, for example, 2D/3D plan beams. We are, therefore, motivated to study an alternative method of producing a flat photon beam without using a FF.

To that end, a seemingly straightforward idea would be to use IMRT methods to generate flat dose maps (perpendicular to the beam axis). To the best of our knowledge, no such attempts have been published. However, our previous study provides some insight as to whether or not current IMRT delivery techniques (e.g., step‐and‐shoot, sliding window) are suitable to achieve such a goal.^14^ Although the study suggests that reasonably flat dose maps can be achieved in most cases, some practical issues still need to be resolved prior to the full implementation of a FFF‐only machine. From one perspective, step‐and‐shoot delivery can generate the best beam flatness with loose segmentation number restriction. However, its delivery efficiency can be as much as five times worse than the conventional beam for the same field size. From another perspective, if the segment number was restricted extensively for the optimization in order to obtain better delivery efficiency, the flatness generated by the modulated FFF beams would become unacceptable for some cases, especially for those with large field sizes. Compared to step‐and‐shoot delivery, the sliding window technique appears to be a more promising delivery method for keeping both flatness and efficiency to a clinically acceptable level, according to our study.^14^ Yet, the currently available SW models fail to incorporate most of the multileaf collimator (MLC) constraints into a global optimization scheme, such as tongue and groove, leaf gap, maximum leaf speed, movable carriage etc.^15^ Instead, they only incorporate MLC constraints at the leaf sequencing step (convert from optimized fluence maps to final deliverable plans). This can largely compromise the Fluence Map Optimization (FMO) results. To be specific for our application, both beam flatness and MLC delivery efficiency of the final MLC converted plan can deteriorate considerably, with respect to the original FMO plan, due to the leaf sequencing step. The detailed results were discussed in our previous study and will not be repeated here. Instead, the interested reader can refer to our original article.

For this study, we developed a direct leaf trajectory optimization (DLTO) model to generate flat beams using a modulated FFF beam with sliding window delivery. Our model incorporates all dynamic MLC constraints into the optimization scheme rather than considering them only during the leaf sequencing process. Delivery efficiency control was included in the optimization model. Tongue and groove effect was also incorporated into the optimization model, as flat beam generation for some concave shape fields can be severely hampered without considering it. With the convexity character of the model, the optimal solution can be guaranteed. The dose map flatness and delivery efficiency were evaluated with the delivery results. The clinical implication and research extension of the proposed model are discussed at the end of the paper.

## 2 MATERIALS AND METHODS

We first describe the machine and MLC characteristics utilized in Section II.A. A general DLTO model is presented afterwards in Section II.B. In Section II.C, we first address the shortfalls of the general model. Then, a new convex model, which incorporates all MLC constraints, is introduced to address the shortfalls. In Sections II.D and II.E, we integrate two additional features, efficiency control and tongue and groove effect control, into the optimization model. For final beam delivery, trajectory map conversion is demonstrated in Section II.F. Finally, the optimization weighting factors, beam delivery and evaluation methods for the flat beam production are described in Section II.G.

### 2.1 FFF beam and MLC characteristics

The FFF beam model of a Versa HD (Elekta Inc. Stockholm Sweden) machine was used for flat beam profile generation. Final dose maps of the modulated FFF beam were compared to its counterpart generated by open FF beams. The energy of the tested beam was 6 MV with a nominal dose rate 600 MU/min for the FF beam and 1400 MU/min for the FFF beam. The unit features the “Agility” head, which has a 160 leaf MLC (80 leaf pairs), and a 0.5 cm leaf‐width projection at isocenter, with a maximum leaf speed of 6.0 cm/s and interdigitating capability. The MLC requires a 6 mm leaf gap for all leaf pairs during dynamic delivery. The leaf extension limit from a movable carriage is 15 cm into the opposing plane. Each bank of the movable carriages cannot pass the center line of the beam. The leaf extension limit for each individual bank is 20 cm, which is also the maximum distance between any two leaves from the same leaf bank.

### 2.2 Direct leaf trajectory model

A two‐step planning strategy has been adopted by most of the commercial treatment planning systems (e.g., Pinnacle (PHILIPS, Holland), Eclipse (VARIAN, Palo Alto, CA) Monoco (ELETKA, Stockholm Sweden) and etc.) for SW‐based IMRT planning. It starts with an optimized FMO obtained by a fluence optimization algorithm, and then converts it to a deliverable leaf sequence by considering all of the leaf constraints using a leaf sequencing algorithm. The leaf sequencing algorithm is similar to the original works proposed by Convery and Rosenbloom (1992), Chui and Spirou (1994), and Convery (1998).^16^, ^17^, ^18^ However, this approach can potentially degrade the original optimized dose distribution generated by the FMO, due to the conversion process, since the optimized fluence map can be compromised by the restricted leaf movement. Such a drawback can severely alter the flatness of the beam for our application. The degradation effect has been thoroughly investigated in our previous study. The desire to mitigate the degradation of the optimal fluence due to the two‐step process led us to pursue a direct optimization approach.

The DLTO model was initially introduced by Papp and Unkelbach (2014) for VMAT optimization.^19^ Rather than optimizing the fluence as the traditional FMO two‐step approach does, DLTO is able to directly model the leaf trajectories in a linear form and use them as the optimization constraints. The leaf trajectory constraints were established based on the arrival/departure time satisfaction of each leaf pair. The convexity of the model is guaranteed by the convexity of the objective function and the linearity of the constraints. The model can be briefly described as follows.

If we let $d_{i}$ represent the absorbed dose to voxel *i* and $d_{i}^{D}$ denote the desired dose to the same voxel, then the objective function ***f*** can be defined as a form of the summation of least square approximation between absorbed dose and desired dose over all voxels, as indicated in Eq. (1) $d_{i}$ can be computed as the summation of the product of the dose influence factor $D_{\mathrm{nij}}$ and beam intensity fluence $x_{\mathrm{nj}}$ over all leaf pairs *n* and bixel location *j*, as shown in Eq. (2). Beam intensity fluence $x_{\mathrm{nj}}$ can be further represented by the product of the constant dose rate *DR* and effective beam‐on time $t_{\mathrm{nj}}$ at bixel j of leaf pair *n,* as shown in Eq. (3), with the assumption that beam intensity fluence, $x_{\mathrm{nj}}$, is proportional to the effective beam‐on time $t_{\mathrm{nj}}$.

(1) $$\text{minimize}\;\;f\left(d\right)=\sum_{i}{\left(d_{i}-d_{i}^{D}\right)}^{2}$$

(2) $$\text{subject}\;\text{to}\;d_{i}=\sum^{N}\sum^{J}D_{\mathrm{nij}}x_{\mathrm{nj}}$$

(3) $$x_{\mathrm{nj}}=DR\cdot t_{\mathrm{nj}}$$

In the above optimization model, variable $d_{i}$ has been successfully transferred to variable $t_{\mathrm{nj}}$, the effective beam on time. $t_{\mathrm{nj}}$ can then be determined by the relationship of the arrival/departure times between the leading leaf and the trailing leaf of the same pair, as indicated in Eq. (4).

(4) $$t_{\mathrm{nj}}=\frac{1}{2}\left[l_{\mathrm{nj}}^{\mathrm{out}}-r_{\mathrm{nj}}^{\mathrm{out}}+l_{n\left(j+1\right)}^{\mathrm{in}}-r_{n\left(j+1\right)}^{\mathrm{in}}\right]$$

**Figure 1:**
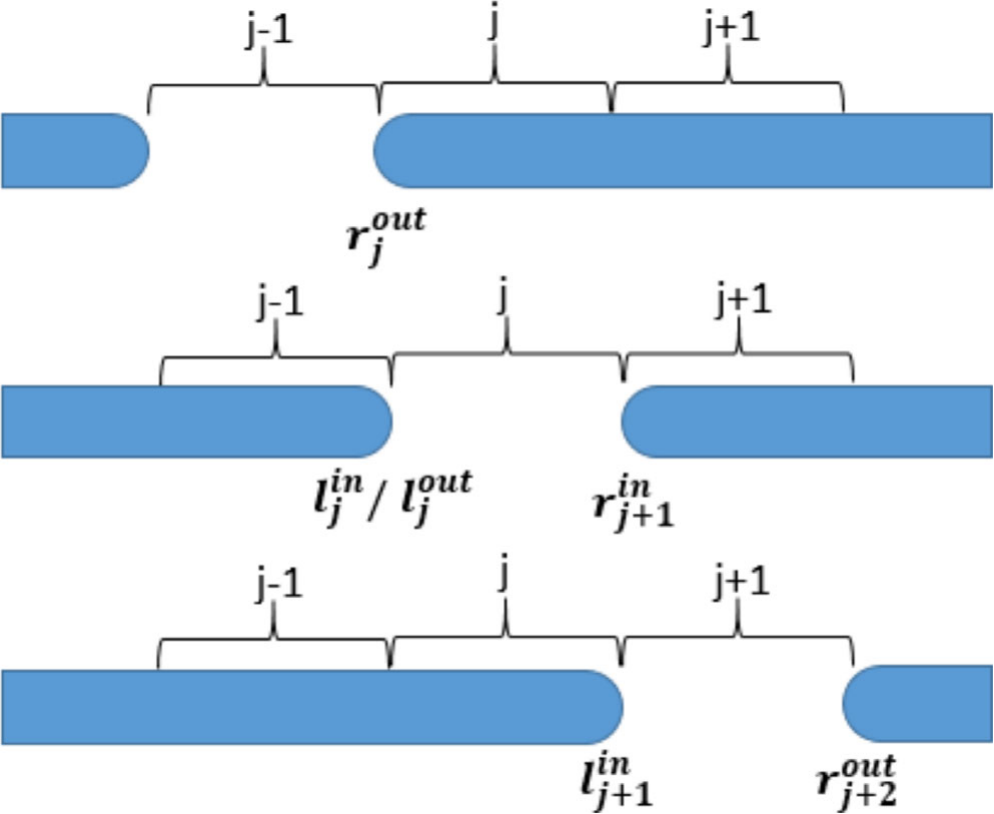
A simple schematic for one leaf pair displaying the trajectory arrival and departure “times” as the leading and trailing leaf traverses one bixel *j* location.

In Eq. (4), $r_{\mathrm{nj}}^{\mathrm{in}}$ and $r_{\mathrm{nj}}^{\mathrm{out}}$ represent the arrival and departure times of the leading leaf at the boundary between bixel ***j‐1*** and ***j*** for leaf pair n, respectively. Similarly, $l_{\mathrm{nj}}^{\mathrm{in}}$ and $l_{\mathrm{nj}}^{\mathrm{out}}$ represent the arrival and departure times of the trailing leaf at the boundary between bixel *j‐1 *and *j* for leaf pair *n*, respectively**.** The arrival and departure times are depicted in Fig. 1, for one leaf pair trajectory, traversing bixel **j**.

To ensure that the breakpoints in the piecewise linear leaf trajectories are properly ordered and the trailing leaf is always behind the leading one, Eq. (5) and Eq. (6) were also added into the constraints.

(5) $$0\leq r^{\mathrm{in}}\leq r^{\mathrm{out}},\;0\leq l^{\mathrm{in}}\leq l^{\mathrm{out}}$$

(6) $$r^{\mathrm{in}}\leq l^{\mathrm{in}},\;r^{\mathrm{out}}\leq l^{\mathrm{out}}$$

These MLC timing constraints enforce monotonic leaf motion in the DLTO model and allow us to directly optimize leaf trajectories, along with finding the optimal dose distribution. This model is a convex optimization problem, as the objective function is convex and the constraints are linear. Convex optimization problems can be solved efficiently, in general, and global optimal solutions are guaranteed.

In principal, DLTO can be applied to the proposed problem to acquire the modulation to achieve a flat beam using the FFF beam. However, the general DLTO model only accounts for MLC travel timing constraints with no consideration of the limits of dynamic delivery, such as leaf gap and leaf travel limits. Therefore, the model is not practical for deliverable plan generation. In addition, the model does not include delivery efficiency and tongue and groove effect in the optimization, which are essential to improving the final delivery efficiency, and flatness, of the dose map, respectively. Thus, to make the DLTO model clinically feasible, an overhaul of the original model is needed. In the next three subsections, we incorporate all of the beam delivery necessities into the model, while keeping the convexity of the problem and the linearity of the constraints intact.

### 2.3 Dynamic MLC delivery constraints

The DLTO model can be extended to take into account the additional dynamic delivery machine constraints while keeping the linearity of the constraints intact. The idea can be described as follows. As variable ***j*** represents the distance and location indicator for each leaf in the trajectory, we can intuitively utilize ***j*** to introduce distance‐based constraints for the MLC. The added constraints include minimum leaf gap, maximum leaf travel distance into the opposing plane, maximum leaf travel of adjacent leaves in the same bank, and equal beam off times for all leaf pairs. Each of these is described separately in this section.

To incorporate the minimum leaf gap of opposing leaves during delivery, denoted as *Lgap*, the general model of Eq. (6) can be rewritten as Eq. (7) to directly enforce the leaf gap requirement. Such a constraint imposes the minimum gap throughout the entire trajectory, and also keeps the trailing leaf behind the leading one. The range of subscript, *j*, in Eq. (7) should be confined by leave’s maximum travel distance across the centerline for both banks. Thus, both the starting bixel *j* $,j_{\mathrm{start}}$ and ending bixel *j,* $j_{\mathrm{end}}$ are restricted to be less than the maximum leaf travel distance, as indicated in Eq. (8).

(7) $$r_{\mathrm{nj}}^{\mathrm{out}}\leq l_{n\left(j-Lgap\right)}^{\mathrm{out}}$$

(8) $$\begin{array}{c} \text{Left}\;\text{Bank}:j_{\mathrm{start}}\leq Travel\;Constr.and\;j_{\mathrm{end}}\leq Travel\;Constr.\\ Right\;Bank:j_{\mathrm{start}}\geq \left(-\right)Travel\;Constr.and\;j_{\mathrm{end}}\geq \left(-\right)Travel\;Constr. \end{array}$$

Equation (9), implements the leaf carriage constraint. The constraints will be enforced only if a potential violation is found due to field shape and size. In that manner, we can customized the size of the constraints for each individual model to acquire better computation efficiency. This is achieved by preprocessing and evaluating the difference in starting and ending locations for each leaf trajectory for a given field shape. A potential violation arises if the difference in starting positons, difference in ending positons, or difference between the start and end positons is greater than the maximum allowed distance. If a potential violation is found, then constraints will be added to the model for each potential. The functions of the introduced restrictions are similar to those of Eq. (8), as it limits the range of the bixel indicator **j**, for a given leaf bank.

(9) $$\begin{array}{c} \mathrm{If}\;\left|j_{S_{n}}-j_{S_{m}}\right|>Carr.Constr.\;\text{or}\;\left|j_{S_{n}}-j_{E_{m}}\right|>Carr.Constr.\;or\;\left|j_{E_{n}}-j_{E_{m}}\right|\\ \mathrm{Then}\;l_{\mathrm{nj}}^{\mathrm{out}}\leq l_{m\left(j+Carriage\;Constr.\right)}^{\mathrm{out}},\;r_{\mathrm{nj}}^{\mathrm{out}}\leq r_{m\left(j+Carriage\;Constr.\right)}^{\mathrm{out}}\;\forall n\neq m \end{array}$$

*S* and *E* in Eq. (9) refer to the starting and ending locations for each leaf pair, respectively. Subscripts *n* and *m* represent the number of any two leaves in the same bank.

By enforcing all trailing leaves “out” time to be equal for the last leaf position, Eq. 10 can guarantee that beam off times for all leaf pairs are the same. The necessity of beam off time constraint arises from the fact that the beam will stop for all leaf pairs at the same time that the last trailing leaf stops moving.

(10) $$l_{n\left(jend-Lgap\right)}^{\mathrm{out}}=l_{m\left(jend-Lgap\right)}^{\mathrm{out}}$$

### 2.4 Efficiency control

To control the delivery efficiency, two methodologies can be applied in the original optimization scheme. One is to restrict total delivery time in a constraint format; the other is to add a weighted delivery time term into the objective function. For this work, we chose the second method since it not only provides the flexibility to control the delivery efficiency by varying the weighting, but also diminishes the issue of an infeasible solution, which is unavoidable with the first methodology.

Since the total treatment time can be expressed as $l_{n\left(j_{\mathrm{end}}-\mathrm{Lgap}\right)}^{\mathrm{out}}$ (the trailing leaf’s out time for the last leaf position), the objective function of Eq. (1) can be rewritten as Eq. (11) to incorporate the delivery efficiency control.

(11) $$\text{minimize}\;f\left(d\right)=\lambda_{1}\sum_{i}{\left(d_{i}-d_{i}^{D}\right)}^{2}+\lambda_{2}l_{n\left(jend-Lgap\right)}^{\mathrm{out}}$$

in which $\lambda_{1}$ and $\lambda_{2}$ are weighting factors for dose uniformity and delivery efficiency, respectively.

### 2.5 Tongue and groove effect

**Figure 2:**
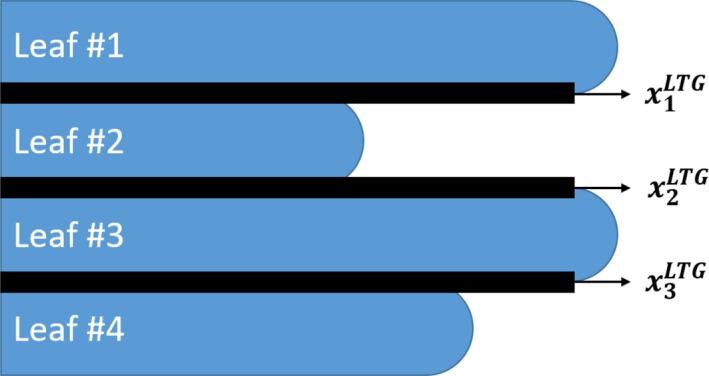
Simple example of beam’s eye view for the left leaf bank, showing the location for tongue and groove fluence calculation. The dark strips between adjacent leaves represents the 1 mm area for fluence calculation.

To incorporate T&G effect into the optimization model, two extra fluence variables, $x_{\mathrm{nj}}^{\mathrm{LTG}}$ and $x_{\mathrm{nj}}^{\mathrm{RTG}}$ **,** corresponding to the T&G fluence for the left and right leaf banks, respectively, were introduced into the model. They represent the partial fluence transmission at T&G edges (1 mm strips) between adjacent leaves from both banks. The geometric location of T&G fluence is shown in a schematic drawing as an example, demonstrated in Fig. 2. Extending the general model, T&G fluence is calculated utilizing two new “effective beam on time” variables for the left and right leaf banks, $t_{\mathrm{nj}}^{\mathrm{LTG}}$ and $t_{\mathrm{nj}}^{\mathrm{RTG}}$, respectively. Thus, Eq. (12) and Eq. (13) are able to approximate T&G effective beam on time for each pair of adjacent leaves. The equations are similar to those of the general model. However, the time associated with T&G effect is determined by the arrival and departure times of the adjacent leaf *in lieu of* the opposing leaf as indicated in Eq. (4).

(12) $$\begin{array}{c} LeftBank:t_{\mathrm{nj}}^{\mathrm{LTG}}=\frac{1}{2}\left[\left(l_{\left(n+1\right)j}^{\mathrm{out}}+l_{\left(n+1\right)\left(j+1\right)}^{\mathrm{in}}\right)-\left(l_{\mathrm{nj}}^{\mathrm{out}}+l_{n\left(j+1\right)}^{\mathrm{in}}\right)\right]\\ x_{\mathrm{nj}}^{\mathrm{LTG}}=DR\cdot t_{\mathrm{nj}}^{\mathrm{LTG}} \end{array}$$

(13) $$\begin{array}{c} RightBank:t_{\mathrm{nj}}^{\mathrm{RTG}}=\frac{1}{2}\left[\left(r_{\mathrm{nj}}^{\mathrm{out}}+r_{n\left(j+1\right)}^{\mathrm{in}}\right)-\left(r_{\left(n+1\right)j}^{\mathrm{out}}+r_{\left(n+1\right)\left(j+1\right)}^{\mathrm{in}}\right)\right]\\ x_{\mathrm{nj}}^{\mathrm{RTG}}=DR\cdot t_{\mathrm{nj}}^{\mathrm{RTG}} \end{array}$$

To minimize the T&G effects, total time associated with T&G effect from both banks was added into our objective function for minimization. The objective function will then be rewritten in the form of Eq. (14)

(14) $$\text{minimize}\;f\left(d\right)=\lambda_{1}\sum_{i}{\left(d_{i}-d_{i}^{D}\right)}^{2}+\lambda_{2}l_{n\left(jend-Lgap\right)}^{\mathrm{out}}+\lambda_{3}\sum_{\mathrm{nj}}\left(x_{\mathrm{nj}}^{\mathrm{RTG}}+x_{\mathrm{nj}}^{\mathrm{LTG}}\right)$$

where $\lambda_{3}$ is the weighing factor tongue and groove effect term.

### 2.6 Trajectory map conversion for beam delivery

**Figure 3:**
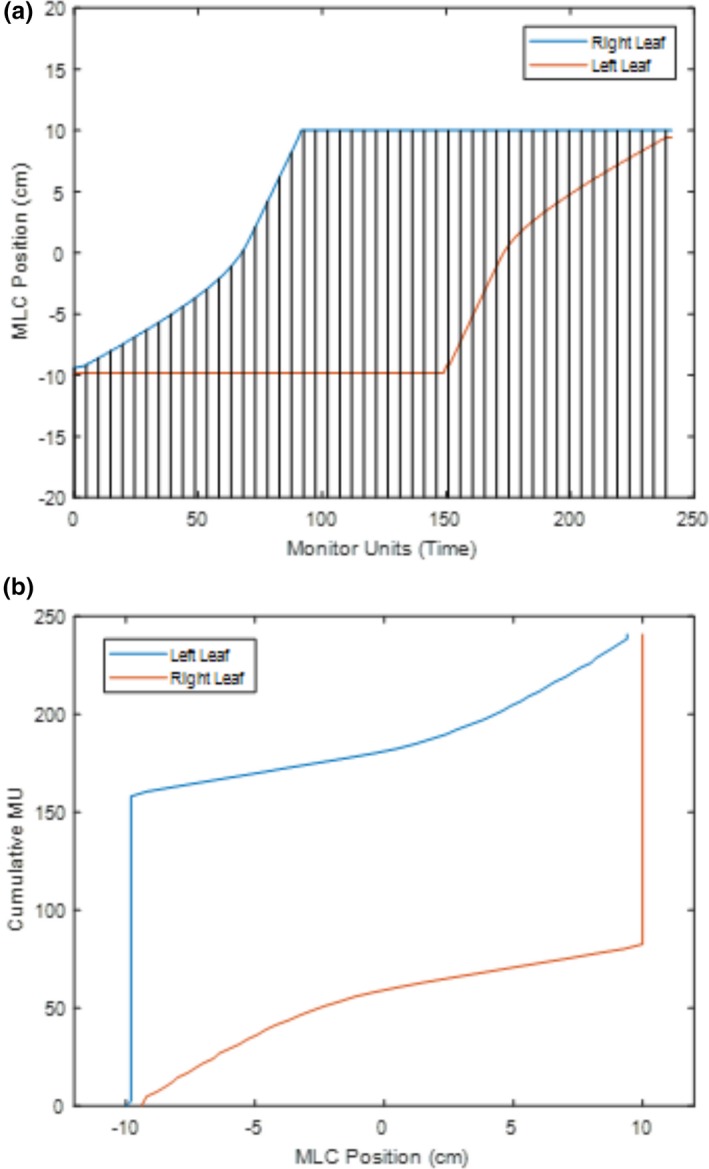
An example of sliding window leaf trajectory for one individual leaf pair for a 20 × 20 cm^2^ field, showing (a) the trajectory before conversion including the control point sampling (50 control points) and (b) the resulting machine deliverable cumulative MU per MLC position.

Prior to the delivery of the modulated FFF beams, the trajectory maps had to be converted from “times” to “positions”. The reason for this conversion is that the final information required for machine delivery is not the arrival/departure time of the MLC at each position, but the MLC positions under certain time/MU intervals. This was handled by sampling the trajectories at equidistant MU (time) intervals and recording the positions. An example demonstrating the equidistant sampling using a leaf trajectory map is shown in Fig. 3. For beam delivery, the cumulative MU and leaf position for each control point were transferred to the machine using DICOM format.

### 2.7 Optimization results and beam delivery assessment

The optimization model was coded under the MATLAB environment (Mathworks Natick, MA) using CVX software, a type of MATLAB software for solving disciplined convex programming. The solving algorithm employs a predictor‐corrector primal‐dual path‐following method.^20^, ^21^ The code was run on a Dell Optiplex 990 (Dell Inc. Round Rock, TX) with an Intel^®^Core™ i5‐2500 CPU @3.30GHz processor (Intel Corporation, Santa Clara, CA) and 8GB of RAM. Optimization times were recorded for all field geometries analyzed in the study.

The 2D planar dose for a conventional flat beam in a virtual water tank was computed with our in‐house software using a pencil beam algorithm,^22^ which was subsequently used as the desired planar dose, $d_{i}^{D}$, in Eq. (1) for the optimization for any specific field, with 1x1mm^2^ resolution. The measured data for 2D dose maps was obtained from the MapCheck2 (SunNuclear Inc. Melbourne, FL) diode array. Absolute dose calibration for the MapCheck2 device, was performed following the guidelines of the Sun Nuclear Absolute Dose Calibration Protocol from the MapCheck2 Reference Guide.^23^ The measurements for profiles were performed using the IC Profiler (SunNuclear Inc. Melbourne, FL). The plane for dose comparison was defined at 10 cm depth with a 100 cm SAD setup throughout the study.

**Figure 4:**
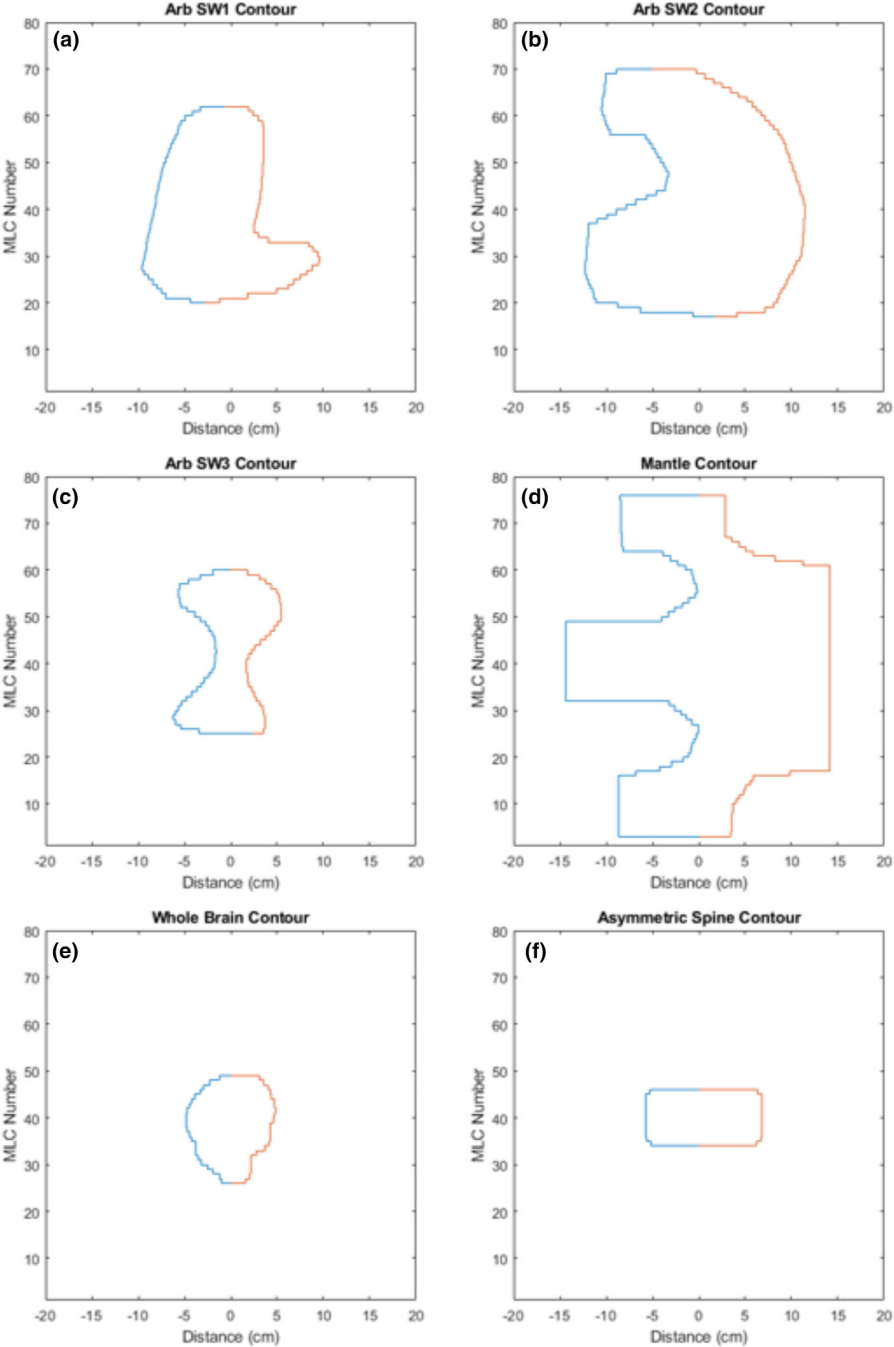
Contours utilized throughout the study, including three arbitrary contours with concave “features”, (a‐c), as well as three clinical contours: (d) mantle field, (e) whole brain field and (f) asymmetric spine field.

The first portion of the assessment focuses on the capabilities of flat beam generation for the fields with a variety of geometries using our optimization model. 2D dose discrepancy between modulated FFF beams and their FF beam counterparts for square fields of various sizes were analyzed using gamma analysis. Clinical fields including whole brain, asymmetrical spine, mantle and three other artificial fields with concave contours were also assessed to test the robustness of the model. All field opening contours are shown in Fig. 4. Tongue and groove effect correction was examined for some special cases. Comparisons between the optimization with and without the tongue and groove effect are listed in the results section to show the efficacy of the proposed method. Delivery time is also listed for all fields used. Computation time was compared for all fields used. The impact of the optimization parameters are evaluated below in the results section.

## 3 RESULTS

### 3.1 Beam flatness assessment

Two dimensional dose comparisons between modulated FFF beams and their FF beam counterparts are presented in Gamma passing rate format in Table 1. The comparison results include all field sizes less than 30x30 cm^2^. The results for field sizes greater than 30 × 30 cm^2^ are not included due to the limited detection area of the MapCheck2 device. Measurement uncertainty is automatically included in the measurement by the device. According the Sun Nuclear MapCheck2 Reference Guide, “The MapCheck2 software calculates a measurement uncertainty for each point and adds it to the acceptance criteria. The uncertainty for absolute dose comparison is around ~0.01.”^23^ The passing rate was obtained utilizing “local” absolute dose comparison with under 3% dose difference and 3 mm Distance‐To‐Agreement (DTA) criteria, with a 10% dose threshold of the central axis dose. The gamma analysis is performed based on the formalism presented by D. A. Low et al.^24^ It can be seen that all fields achieved 100% passing rates, except for the 30x30 cm^2^ field, which has a 99.8% passing rate.

**Table 1:** Gamma comparison passing rates using 3%/3 mm criteria for modulated FFF beams and open FF reference beams for various field sizes.

Field	Gamma passing rate(%) 3%/3 mm
10 × 10 cm^2^	100.0
Spine 15 × 6 cm^2^	100.0
20 × 20 cm^2^	100.0
30 × 30 cm^2^	99.8
Arb SW1	100.0
Arb SW2	100.0
Arb SW3	100.0
Whole Brain	100.0

FFF, flattening filter free.

All fields were measured using MapCheck2 device with 100 SAD and 10 cm buildup set‐up.

**Figure 5:**
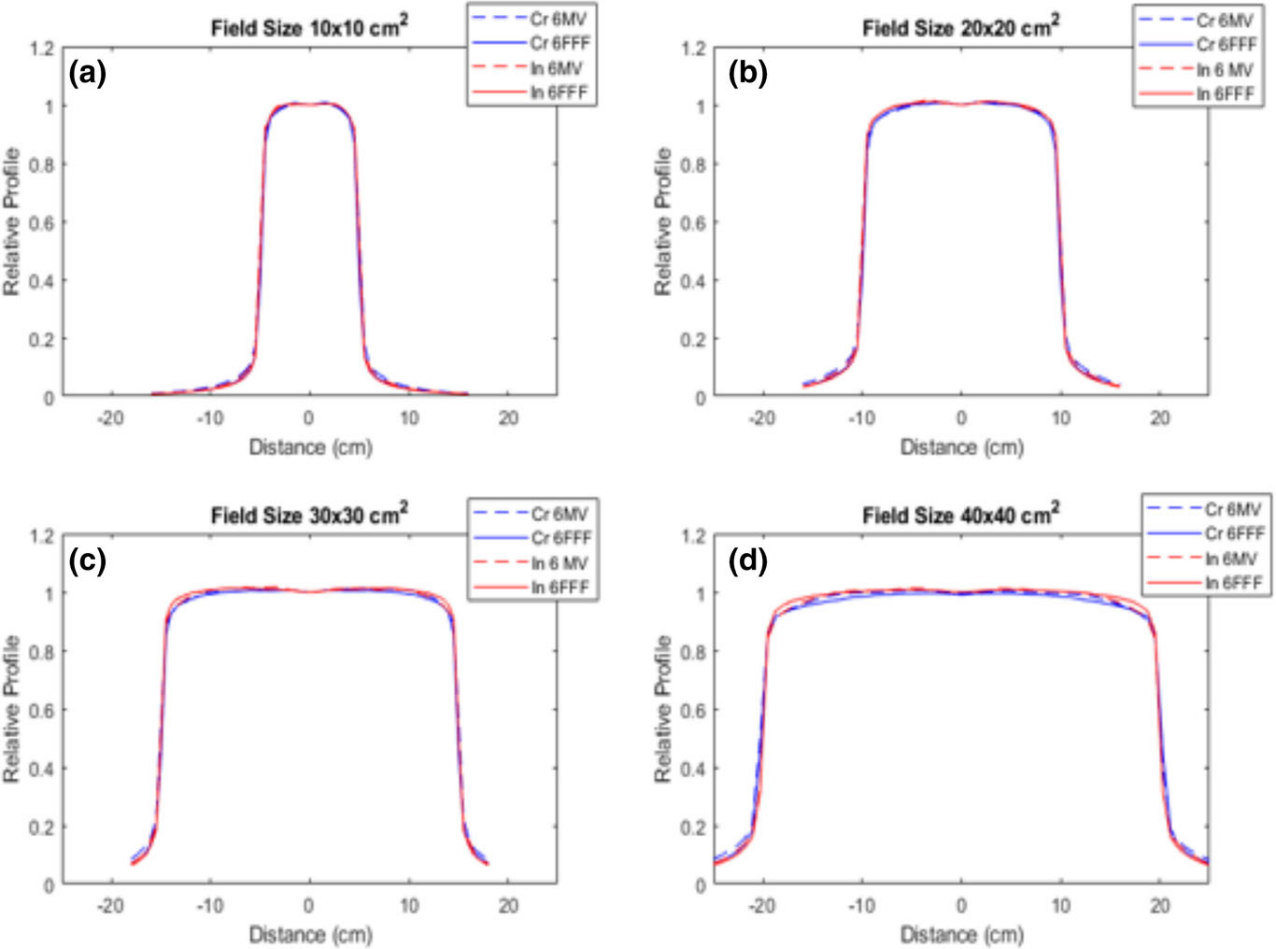
Measured crossline and inline central axis profiles for both modulated FFF and corresponding reference FF beams, field sizes include: (a) 10 × 10 cm^2^, (b) 20 × 20 cm^2^, (c) 30 × 30 cm^2^, and (d) 40 × 40 cm^2^. FFF, flattening filter free.

**Figure 6:**
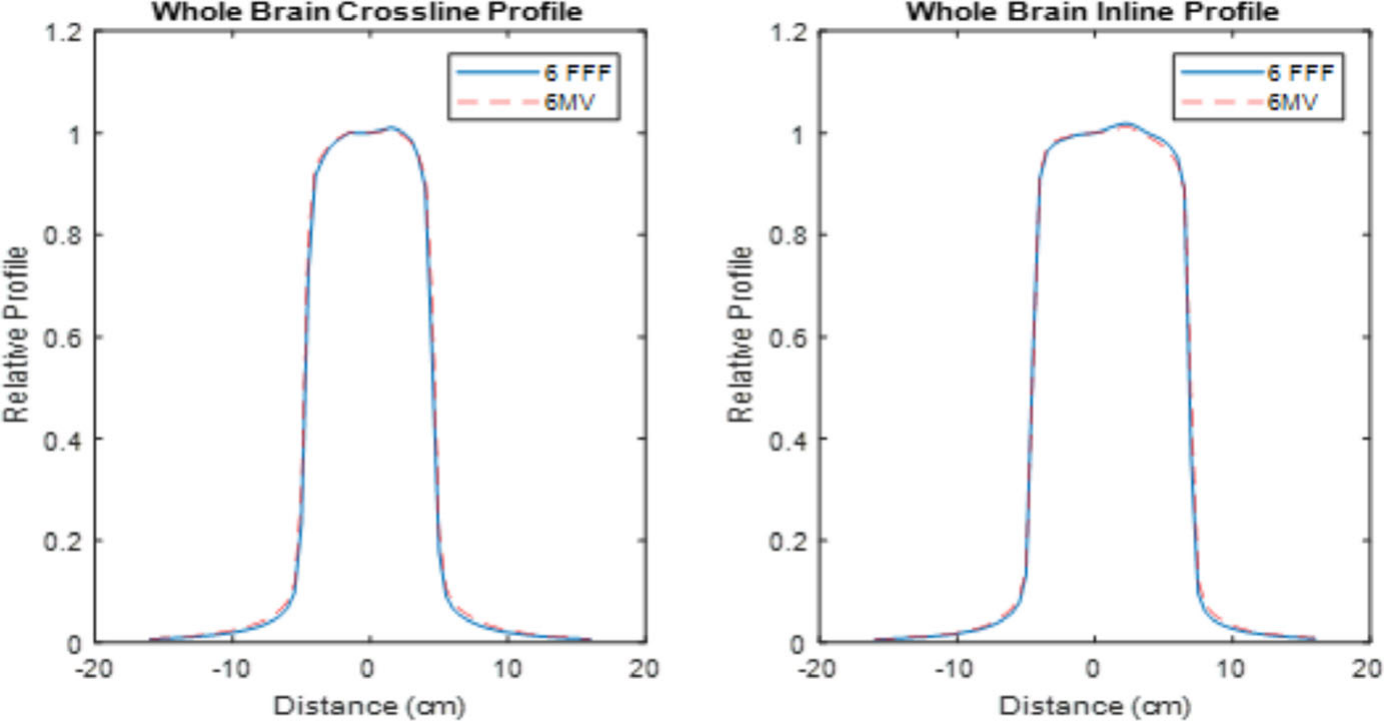
Measured central axis crossline and inline normalized dose profiles for modulated FFF and FF reference beams for a whole brain contour.

**Figure 7:**
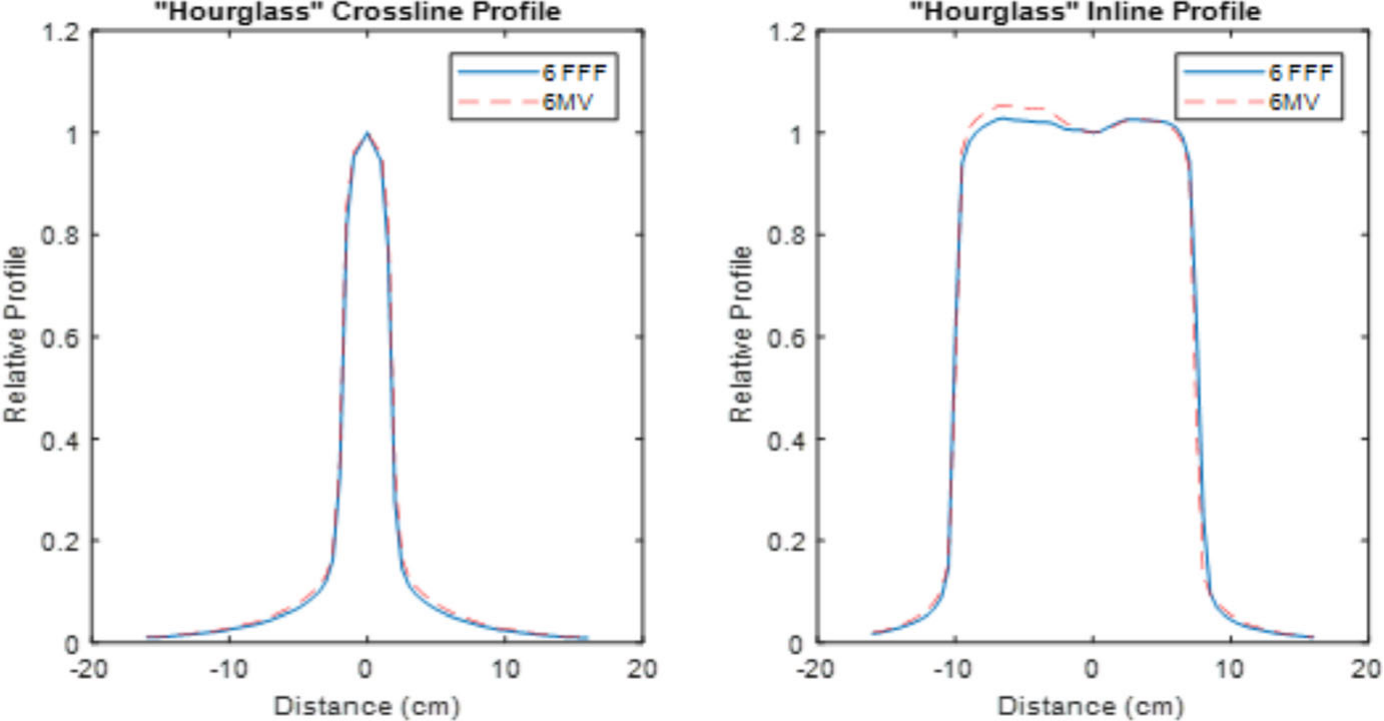
Measured central axis crossline and inline normalized dose profiles for modulated FFF and FF reference beams for an arbitrary “hourglass” contour (ArbSW#3). FFF, flattening filter free.

The quantitative flatness of the measured central axis profiles for both modulated FFF beams and their corresponding reference FF beams are shown in Table 2. The definition of flatness is in accordance with IEC60976.^25^ The relative central axis profiles are shown in Figs. 5, 6, and 7. Figure 5 displays the profile comparisons for both the crossline and inline for square fields of 10 × 10 cm^2^, 20 × 20 cm^2^, 30 × 30 cm^2^, and 40 × 40 cm^2^. Figures. 6 and 7 show the profile comparisons for the whole brain contour and an arbitrary concave “hourglass” contour shape, respectively.

**Table 2:** Quantitative flatness assessment for measured dose profiles along the crossline and inline of the central axis for modulated FFF beams and reference FF open beams.

Field	FFF Cr‐Plane	FFF In‐Plane	FF Cr‐Plane	FF In‐Plane
10 × 10 cm^2^	2.9	2.5	3.0	3.8
15 × 6 cm^2^	2.7	3.3	3.3	3.9
20 × 20 cm^2^	2.8	2.5	3.1	4.0
30 × 30 cm^2^	2.6	2.4	3.2	4.3
40 × 40 cm^2^	3.6	3.6	3.7	3.8
Arb SW1	3.4	3.3	3.8	3.4
Arb SW2	2.6	3.4	3.5	3.1
Arb SW3	2.8	2.9	3.1	5.4
Whole Brain	3.1	2.8	3.9	4.3
Mantle	6.4	3.5	4.7	4.5
Mantle T&G*	3.8	3.4	4.7	4.5

FFF, flattening filter free.

* The field was optimized including the tongue and groove fluence model, in the objective function.

### 3.2 Tongue and groove effect correction

**Figure 8:**
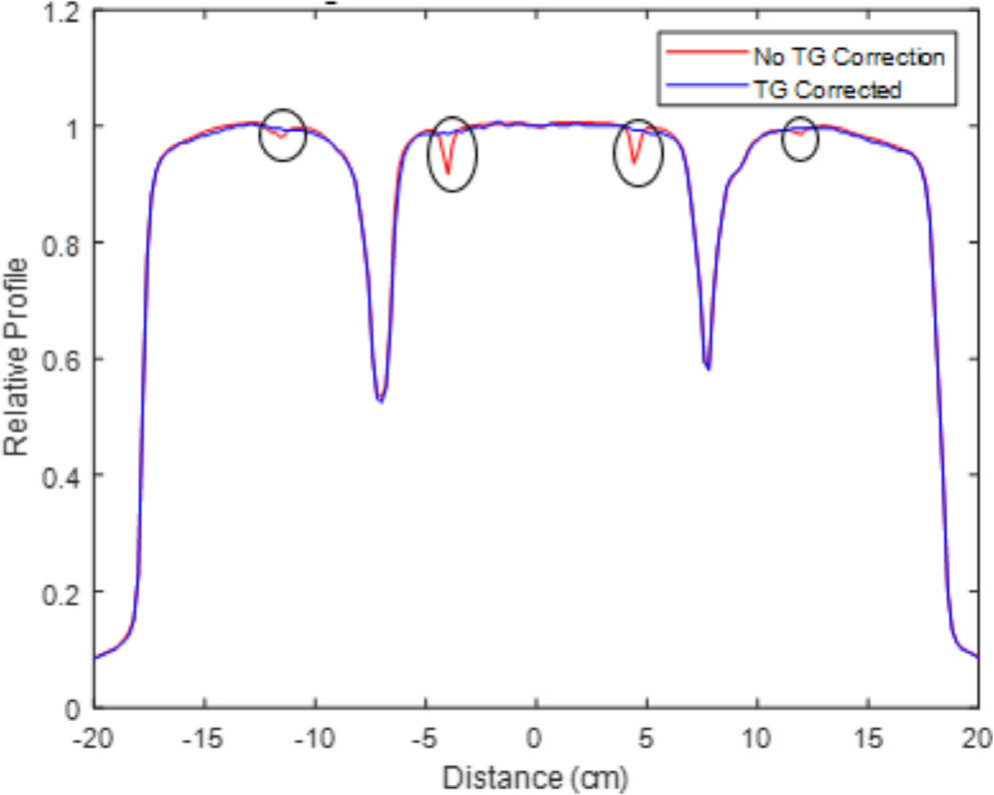
Central axis crossline normalized dose profile for a modulated FFF mantle field. The comparison of the two profiles highlights the ability to eliminate the profile dips attributed to the tongue and groove effect (circled). FFF, flattening filter free.

The T&G effect control scheme described in Section II.E was employed for the fields with severe T&G effect after the initial modulation, for example, the mantle field. Figure 8 illustrates the crossline central axis profile difference of a mantle field with and without T&G effect correction. The flatness of the T&G effect‐corrected mantle field is also recorded in Table 2.

### 3.3 Treatment time comparison

Delivery time comparisons between modulated FFF beams and their FF open beam counterparts are shown in Table 3. It is important to note that the nominal dose rate of the FFF beams is 1400 MU/min, which is much higher than nominal dose rate of the FF beam of 600 MU/min. Table 3 indicates that the method can achieve better delivery efficiency for field sizes of less than 15 × 15 cm^2^, compared to FF open beams. For larger field sizes, our delivery efficiency can become worse and the delivery times are longer than for conventional deliveries. The maximum ratio of the delivery time between the modulated FFF and the open FF beams is 2.5, which corresponds to an additional 33.23 s of delivery time.

**Table 3:** Total treatment delivery time for FFF modulated flat beams and reference FF static beams, for all fields delivered.

Field	FFF (seconds)	FF (seconds)	FFF/FF
10 × 10 cm^2^	23.40	26.0	0.90
20 × 20 cm^2^	32.03	23.9	1.34
30 × 30 cm^2^	44.00	23.0	1.91
40 × 40 cm^2^	55.23	22.0	2.50
Arb SW1	28.14	25.0	1.13
Arb SW2	34.19	23.9	1.43
Arb SW3	22.99	26.7	0.86
Whole Brain	17.20	23.8	0.73
15 × 6 cm^2^	21.56	26.2	0.82
Mantle	33.80	22.6	1.49
Mantle T&G*	39.42	22.6	1.74

Note

FFF, flattening filter free.

For modulated FFF beams the max dose rate is 1400 MU/min, for FF beams the max dose rate is 600 MU/min.

* The field was optimized including the tongue and groove fluence model, in the objective function.

### 3.4 Computation assessment

Computation times for modulation generation were compared for all cases presented in the study, and are displayed in Table 4. The table includes overall calculation times and specific interior point optimization times.^26^ For all fields tested, except for the 40 × 40 cm^2^ square field, the computation times are less than 30 s. The 40 × 40 cm^2^ square field takes 80 s for optimization. The mantle field computation with the T&G correction requires a bit more computation time than the same field without T&G correction.

**Table 4:** Optimization time to generate the MLC modulation for all fields measured throughout the study.

Field	Calculation Time (s)	Interior point time (s)
10 × 10 cm^2^	16.032648	0.95
20 × 20 cm^2^	19.316858	1.37
30 × 30 cm^2^	25.352494	1.98
40 × 40 cm^2^	80.154531	2.28
Arb SW1	19.063774	1.28
Arb SW2	21.735680	1.89
Arb SW3	17.556734	1.15
Whole Brain	16.590719	1.06
15 × 6 cm^2^	24.759497	1.12
Mantle	28.209213	2.29
Mantle T&G*	34.294071	5.54

* The field was optimized including the tongue and groove fluence model, in the objective function.

**Figure 9:**
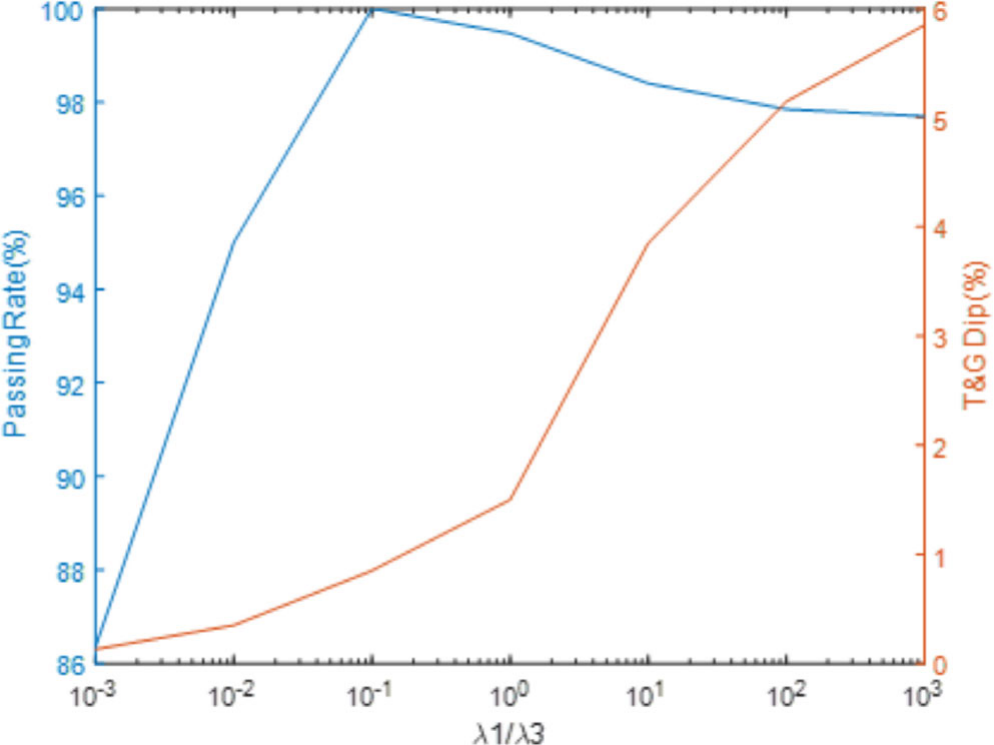
Specifically for the mantle field, an evaluation of the tradeoff between Gamma passing rate and tongue and groove effect, due to the optimization weighting factors λ1 and λ3, the flatness control term and the tongue and groove control term, respectively.

**Figure 10:**
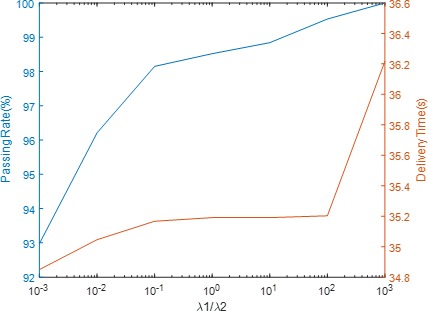
An evaluation of the tradeoff between the Gamma passing rate and the delivery time, averaged across all fields analyzed, due to the optimization weighting factors λ_1_ and λ_2_, the flatness control term and the delivery time control term, respectively.

The correlations between the weighting factors of Eq. 14 and delivery performance are displayed in Fig. 9 and Fig. 10. Figure 8 depicts the trend lines of the gamma passing rate and the relative “dip dose” difference (indicated in Fig. 8), along with the λ_1_/ λ_3_ ratio variation for the mantle field. Figure 10 illustrates the trend lines of the Gamma passing rate and delivery times along with the λ_1_/ λ_2_ ratio variation for the mantle field.

## 4 DISCUSSION

All modulated flat beams exhibited comparable flatness to FF open beams, as indicated in Table 1, Table 2 and Figs. 5, 6, 7, 8. 2D measurement results (Gamma passing rates summarized in Table 1) demonstrate that the dose differences between modulated FF beams and their corresponding FF open beams are minimal for all field sizes tested. This suggests that our modulation method not only performs well for fields with regular field shapes, but also properly functions for fields with irregular field shapes. The 1D dose profile comparison results (quantitative flatness summarization in Table 2) present a similar indication. It is also worth pointing out that the profile discrepancies between the modulated FFF beams and their corresponding FF beams is clinically acceptable, while still being qualitatively apparent on measurements, especially for fields with irregular shapes (e.g., Arb SW3 field). Figure 7(b) provides an example of an obvious discrepancy between the two profiles (left shoulder region of the profile). Such a discrepancy is mainly due to the approximation applied in Eq. (3) of the optimization model. In Eq. (3), we assumed that the relative beam intensity of any beamlet is proportional to the effective beam‐on time of that same beamlet. In reality, the true intensity is determined not only by beam‐on time, but also by the in‐air scatter conditions for that beamlet, which are a result of collimation shape, size and position. As long as the beam aperture varies during the delivery, the relative beam intensity will deviate from the beam‐on time factor assumption to some degree. This deviation can cause an inconsistency between the optimized fluence and the true fluence, and ultimately lead to a discrepancy between the modulated dose profiles and open beam profiles. Since the irregular fields typically require more complicated modulation to achieve flatness, the discrepancy can become more profound, when compared to regular fields, as significant aperture variation can take place during the SW delivery due to the modulation complexity. Nevertheless, our current method can still generate profiles with a clinically acceptable flatness and a clinically insignificant discrepancy between modulated FFF beams and FF open beams.

Fluence transmission associated with the T&G design was successfully included in our optimization model to minimize the T&G effect. With the T&G effect minimization model, the dose profile “dips” have been completely removed from the profile of the mantle field, as indicated in Fig. 8. The measured quantitative degree of flatness in Table 2 implies a comparable result. Since the T&G effect control term was added into the objective function (the original objective function only has the flatness control term), the weighting factors for the flatness control term and the T&G effect control term need to be properly weighted to achieve an optimal trade off. According to Fig. 9, the optimal clinical value of the λ_1_ over λ_3_ ratio should be chosen to be in the range of 0.1 to 1 in order to maintain flatness while minimizing the dose profile “dips” attributed to the T&G effect. Our final result for the mantle field uses a value of 0.1 as the ratio of λ_1_ over λ_3_, optimized with the T&G effect control term in the model. An interesting observation is that the T&G effect is only noticeable in the irregular concave‐shaped field contour (e.g., Mantle field). For the majority of the regular fields, the T&G effect is negligible, even when the beams are optimized with the absence of the T&G effect control term in the objective function.

As discussed in the introduction, beam flatness is not the only criteria that can be used to evaluate our method. Delivery efficiency is another key factor to assess the feasibility and practicality for the beam delivery. According to Table 3, the delivery times of modulated FFF beams are actually slightly shorter than their open FF counterparts for field sizes less than 15 × 15 cm^2^, whereas for larger field sizes slightly longer delivery times for modulated FFF beams are observed. However, the increase in delivery times for the larger fields falls within a reasonable range of less than 2.5 times that of their open FF counterparts for all fields but the 40 × 40 cm^2^ field. The comparable modulated FFF to open FF delivery time makes our method practical for clinical implementation. The good delivery efficiency can be attributed to the benefits associated with dynamic delivery, the increased dose rate capability of FFF beams, and the addition of the delivery efficiency control term to the original objective function. As matter of fact, modulation complexity can also be reduced by the introduction of the delivery efficiency control term, as better efficiency generally results in simpler modulation. This advantageous feature can help the algorithm decrease the MLC aperture variation during delivery and ultimately reduce the discrepancy between modulated and open beam profiles, as was discussed previously. Figure 10 suggests that the weighting of the efficiency control term in the objective function can be very low when compared to the flatness control term, as the variation of average delivery time across all weighting schemes is very small, at just 1.7 s. Just the inclusion of the delivery efficiency control term in the objective function provides the benefit of simpler modulation and shorter delivery time. We chose 1000 as the λ_1_ to λ_2_ ratio for all of the cases that were tested.

Regarding the computation efficiency of the optimization algorithm (as shown in Table 4), all total calculation times fall within 35 s, except for the 40 × 40 cm^2^ field size. The computation times (interior point time) are no greater than 6 s for all cases. The model with the T&G effect control required a slightly longer computation time, as it makes the model more complicated to solve. Overall, the calculation time is practical for a time sensitive clinical practice that may, for example, utilize same‐day patient simulation and treatment.

Pertaining to the clinical implementation and future outlook for our model, a few features may present certain clinical advantages. First, the model provides the ability to deliver modulated FFF beams slightly faster than their open FF counterparts for field sizes less than 15 × 15 cm^2^, as shown in Table 3. Faster delivery times can potentially improve motion management for specific treatments. For example, the breath hold technique has been widely used for 3DCRT‐based lung and left breast treatments for tumor motion control. Using a modulated FFF beam can decrease patient breath hold time. Second, the ability to plan conventional treatments independent of the treatment planning system provides for the possibility of application to time sensitive clinical cases, such as cord compression treatment. Lastly, we believe the structure of our optimization model can also be easily extended to standard SW based IMRT, or even VMAT, optimization. Since our model is able to encompass all possible MLC constraints in linear form, better computation efficiency for direct SW based IMRT and VMAT optimization is possible using our model structure.

## 5 CONCLUSION

We have developed a SW modulation model to generate conventional flat beams while operating in FFF mode to achieve a machine design that permits the complete removal of the flattening filter. The modulation model builds upon the work of Papp and Unkelbach (2014), but it innovatively incorporates all dynamic MLC constraints into the optimization in linear form. Delivery efficiency and tongue and groove effect control are also incorporated into the model. With the convexity character of the model, a global optimal solution can be guaranteed. The results presented in this work indicate that our model is capable of generating flat modulated FFF beams that are comparable in all aspects to conventional open FF beams. The treatment time and planning calculation time are both acceptable for clinical practice.
